# Does movement preparation enhance attending to bodily sensations in the back in people with persistent low back pain?

**DOI:** 10.1371/journal.pone.0300421

**Published:** 2024-04-18

**Authors:** Amanda Clauwaert, Eleana A. Pinto, Stijn Schouppe, Lieven Danneels, Jessica Van Oosterwijck, Stefaan Van Damme

**Affiliations:** 1 Department of Experimental-Clinical and Health Psychology, Ghent University, Ghent, Belgium; 2 SPINE Research Unit Ghent, Department of Rehabilitation Sciences, Faculty of Medicine and Health Sciences, Ghent University, Gent, Belgium; 3 Pain in Motion International Research Group, Departments of Human Physiology and Rehabilitation Sciences, Faculty of Physical Education & Physiotherapy, Vrije Universiteit Brussel, Brussels, Belgium; Ningbo University, CHINA

## Abstract

Attention has been proposed to play an important role in persisting pain, with excessive attentional processes towards pain information leading to worse pain outcomes and maladaptive behaviors. Nevertheless, research on somatosensory attending during the anticipation of pain-related movements is still scarce. This study investigated if individuals with chronic and recurrent lower back pain compared to pain-free controls, show enhanced attending to somatosensory information in the back while anticipating back-recruiting movements. 43 healthy control, 33 recurrent (RLBP) and 33 chronic low back (CLBP) pain sufferers were asked to perform back-recruiting movements. Before the movement initiation cue, a task-irrelevant tactile stimulus was administered to participants’ lower back to elicit somatosensory evoked potentials (SEPs), used as an index of somatosensory attending. In contrast to our hypothesis, most identified SEP components did not differ across groups. The only exception was the P175 amplitude which was larger for the CLBP group compared to individuals with RLBP and healthy controls. The current study did not find robust evidence of enhanced somatosensory attending to the back in people with persisting lower back pain. The finding that CLBP, but not RLBP individuals, had larger amplitudes to the P175 component, is discussed as possibly reflecting a higher state of emotional arousal in these patients when having to prepare the back-recruiting movements.

## Introduction

Heightened attending to pain-related information is assumed to be a key factor in the development and maintenance of chronic pain [1, 2]. For example, the fear-avoidance model (FAM) states that acute pain might become chronic when increased levels of movement-related fear of pain and attention to bodily sensations lead to excessive avoidance behavior [3, 4]. Empirical evidence on the role of attention in chronic pain comes mainly from studies using paradigms assessing attention to visual pain information (words/pictures; [5–9]). Although meta-analyses suggest that patients with chronic pain attend more to pain information than controls [10–12], it could be questioned if visual pain stimuli are suitable to adequately capture the phenomenon of increased attending to bodily sensations. Although somatosensory attention paradigms have been developed in the pain field [13], research in people with persistent low back pain is still scarce. It was shown that electrocutaneous stimuli on the arm interfered with performance on a tone discrimination task in chronic low back pain (CLBP) patients, particularly in high pain catastrophizers [14]. Moreover, patients with CLBP reporting either high or low pain-related fear, were asked to detect electrical stimuli (administered on the back) and auditory stimuli [15]. They found that the high-fear group was slower in detecting the auditory stimuli, which suggests that fearful patients might tend to prioritize somatic information at the expenses of other attention-demanding information.

In the current study, we aimed advancing this earlier work in several ways. First, pain-related attention is context-dependent and it is more likely triggered by events that are relevant for pain such as movements [3, 16, 17]. Therefore, it is important to assess pain-related attending in more dynamic contexts such as during movement tasks. Second, because reaction times only provide a course index of attending, measuring cortical responses to somatosensory information might yield new insights in attention mechanisms [18, 19]. Third, giving participants the instruction to respond as quickly as possible to a stimulus might contaminate attentional processing. Therefore, by using cortical responses to task-irrelevant somatosensory stimuli, a purer measure of attentional processes can be obtained. Fourth, understanding the role of somatosensory attending in pain chronicity by comparing subgroups of patients with back pain, such as those with chronic and recurrent low back pain (RLBP), could be more insightful [20]. More specifically, CLBP is defined as a pain condition that lasts for at least three months [21], while RLBP suffer from 24-hours flares of pain followed by one month of pain free episodes [22]. In this study, cortical activity related to somatosensory processing was examined via Somatosensory Evoked Potentials (SEPs). SEPs were elicited by task-irrelevant tactile stimuli administered on the back during the preparation of a back-recruiting movement in healthy controls (HC) and patients with CLBP and RLBP. Previous work showed that anticipating experimental back pain while executing back-recruiting movements enhances SEPs to task-irrelevant tactile stimuli administered on the back (i.e., N95 and P166), but no differences emerged between CLBP, RLBP patients and HC [23]. Probably, experimental pain induced similar threat obscuring potential differences across groups. In the current study, differences between groups in somatosensory attending were examined in a natural context. SEPs were obtained during the preparation of a rapid arm movement that required participants to recruit the lower back to maintain the posture. We expected that performing back-recruiting movements would induce threat in CLBP, and possibly in RLBP, but not in HC, resulting in increased amplitude of the SEPs in persistent pain samples. This would indicate that people with persistent back pain are more attentive to the back than pain-free controls during movement preparation. Furthermore, given the difference in chronicity, we expect larger amplitude of the SEPs for the CLBP relative to the RLBP group. Finally, based on the pivotal role that fear and catastrophizing play in pain chronicity [24], we explore associations between attention-related SEPs and pain catastrophizing as well as fear of movement/(re)injury.

## Method

### Recruitment

Participants were recruited via social media, flyers, several Belgian hospitals, and private practices. Pregnant women and individuals with (a history) of respiratory, orthopedic, neurological, systematic, metabolic or circulatory conditions, spinal surgery, spinal trauma or spinal deformities were not eligible for inclusion. Furthermore, only Dutch speaking participants between the ages of 18 and 45, with a healthy body mass index (BMI ≤ 25), left or right-handed were eligible. Importantly, the 3 groups were matched on sex and age.

To be recruited as LBP patient, participants had to suffer from non-specific LBP, which was defined as pain in the lumbar region that is not attributable to a known pathology (e.g. histories of spinal traumata or deformities, degenerative changes or scoliosis, osteoporosis, obesity, radicular signs, malignancies, metabolic or rheumatologic diseases, spinal surgery, neuropathic pain, etc…[25]). The LBP had to be initiated at least ≥ 3 months ago, lasting for at least 24 hours, interfering with daily activities and a medical doctor or physiotherapist had been consulted [22, 26]. Individuals were classified as CLBP when the pain complaints were present on a weekly base, occurring at least on 3 out of 7 days [20]. Individuals were classified as RLBP when they experienced at least 2 reoccurring LBP flares per year during which the mean LBP intensity was rated ≥2 on a visual analogue scale (VAS) [26, 27]. Painful episodes were required to be alternated with pain-free episodes of LBP lasting ≥1 month [26, 28]. RLBP patients were examined only in a pain-free remission period. Participants were included in the control group if they did not suffer from any pain disorders in the past nor had any pain complaints at the time of testing. Moreover, they reported to have never experienced LBP complaints >24 hours that required medical assistance [22, 29–31].

This study focused on movement-induced pain and was part of a larger project. Participants attended two experimental sessions, one involving experimental pain and the other focusing on back-recruiting movements. The present manuscript reports data from the movement-induced pain session, while the other session’s data were published separately [23, 32]. The sessions were scheduled at least 5 days apart and the order was counterbalanced. Participants were asked to refrain from consuming caffeine, alcohol, nicotine and physical exertion 48 hours before or on the day of the experiments. Moreover, participants were asked not to take in painkillers, muscle-specific or general relaxant medication and to maintain a normal sleep pattern the night before testing.

Participants provided informed written consent prior to their inclusion in the study and received monetary compensation and a thorough debriefing after the study. The study was approved by the committee of medical ethics of Ghent University (2016/0168) and adhered to the ethical standards of the declaration of Helsinki.

### Participants

A total of 111 individuals were invited to the lab (35 RLBP, 33 CLBP, and 43 HC). In the RLBP group, 3 individuals were excluded (1 dropped out, 1 had pain during testing, and 1 had BMI ≥ 30). In the CLBP group, 4 participants dropped out and 3 had data recording issues. For the HC group, 7 individuals had data recording issues and 1 fainted during testing. The study was completed by 93 participants. Data collection was carried out in the period between May the 25^th^ (2016) and September the 1^st^ (2019).

### Materials

#### Tactile stimulus

A resonant-type tactor (C-2 TACTOR, Engineering Acoustics, Inc., Florida) was used to administer vibrotactile stimuli (200 ms) to the low back at the L3 spinous process level. The tactor was attached directly to the skin surface by means of a double-sided tape ring and was driven by a custom-built device at 200 Hz. The amplitude and frequency were controlled by a self-developed software program. Since the tactile stimulus creates a small vibrating sound, therefore participants were asked to wear earplugs during the testing.

#### Sensor-box

To register the start of the movement execution, a custom-built optical sensor-box was used. This sensor-box was attached to the participant’s leg at the side of the dominant arm, at a height which the participants could easily reach with fingertip. The role of the sensor box was to record the exact time the movement was initiated in order to obtain a measurement of RT. The device was custom-built by the research support office of our faculty. It consisted of a simple box in which four optical sensors sent a different code when being covered (numbers 1, 2, 3 and 4) and uncovered (numbers 5, 6, 7, and 8). The box used Vishay CNY70 type of optical sensors for response detection. The CNY70 comprised a reflective sensor consisting of an infrared emitter and a phototransistor. The whole sensor was housed in a leaded package that effectively blocked visible light. The box was connected to the recording computer via USB cable (serial COM port) and operated under Arduiino drivers. More info regarding this device is available here: https://rsolabblog.wordpress.com/2016/08/12/osb/.

#### EEG

Brain activity was recorded continuously using the eego sports (ANT neuro system) recording system at a sampling rate of 2,000 Hz from 32 active electrodes, placed according to the international 10/20 setting. The ground electrode was located in the active-shield cap fronto-centrally between the FPz and the Fz electrode. Impedances were kept below 10 kΩ. Further preprocessing was done off-line by using Brainvision Analyzer 2.1 (Brain Products GmbH, Munich, Germany).

#### Experiment software

The experiment was programmed in C-language using the Tscope 5 library package [33]. Triggers were controlled by the experimental software and sent through a custom-made device which allowed to send triggers to the EEG system.

### Procedure

Participants stood straight in front of a computer, with feet shoulder-width apart and arms relaxed by their sides. The screen was positioned 2m away at eye level. They learned the correct arm movement by following instructions from an experimenter. The movement involved either stretching the dominant arm forward to 90° shoulder flexion or moving it backward to a 30° angle of shoulder extension, returning quickly to a sensor box placed at the hip [34, 35]. These *rapid arm movements* disrupted trunk posture, triggering a lower back muscle response to restore balance [36, 37]. Electromyography (EMG) data were collected for arm and trunk muscles but not reported here as they were published elsewhere [32]. During the practice phase, participants performed the movement and received feedback on accuracy and speed for 6 trials (3 in each direction). Practice phase was repeated until the movement was executed as requested.

Then, the experiment started with a practice block of 24 trials (not included in the analyses). Each trial started with the presentation of a fixation cross (200-500ms), followed by a visual warning cue (a white ball) presented for 3000ms in the center of the screen. In this time period, A 500ms vibrotactile stimulus was applied to the lower back to induce somatosensory evoked potentials. The stimulation occurred between 2000 and 2500ms after the cue. After the warning cue vanished, a second cue (Go/No Go cue) indicated the required arm movement. Participants were instructed to execute as quickly as possible a forward or backward arm movement when an upwards (1/4 trials) or downwards (2/4 trials) pointing arrow was presented, respectively. If the cue "STOP" appeared, participants were instructed not to move to prevent arm fatigue. Response latencies were defined as the time between movement cue (one of both arrows) onset and the release of the sensor box.

**Fig 1:**
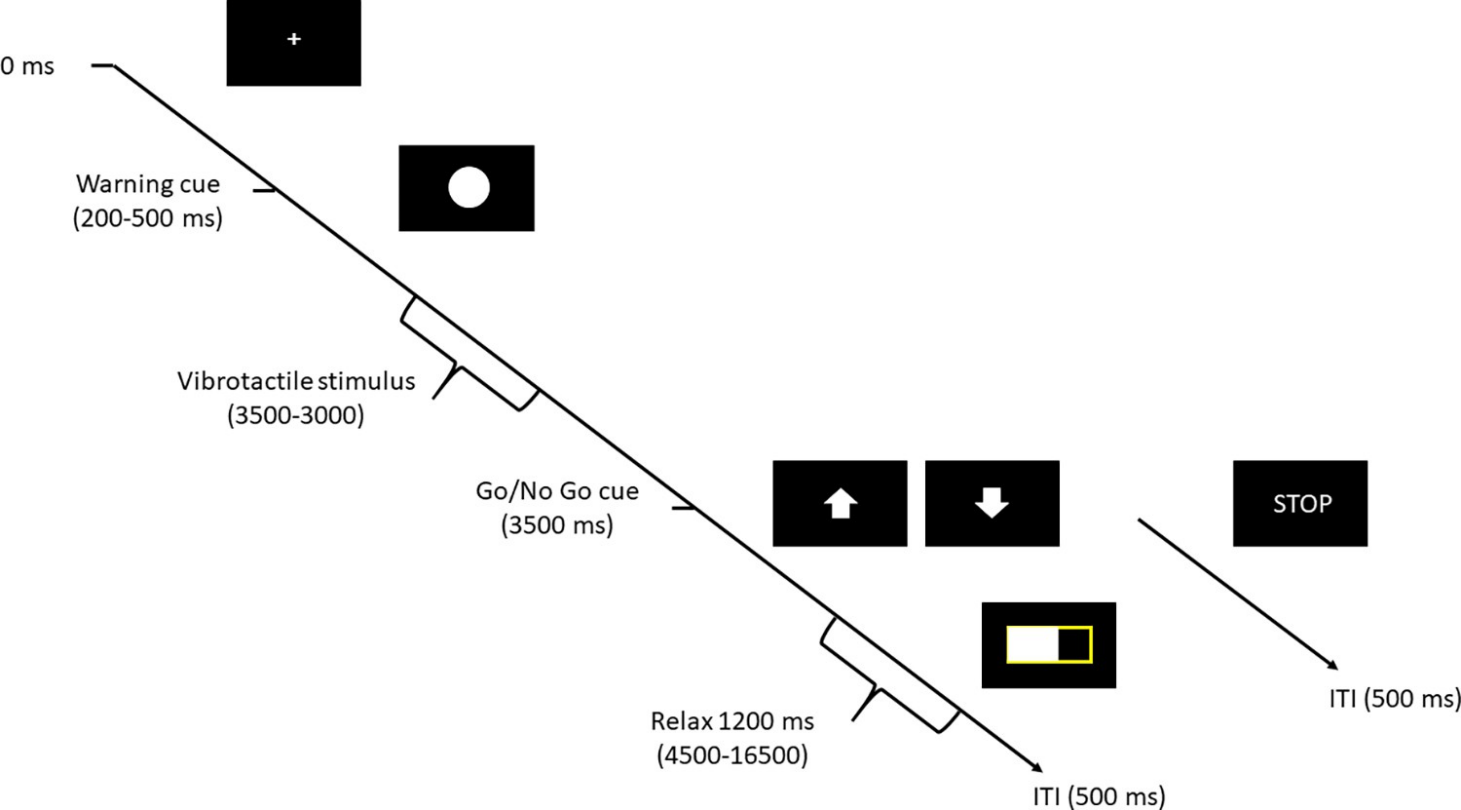
Overview of one experimental trial. Trials started with a warning cue (200–500 ms), after which an innocuous tactile stimulation was administered to the back. Subsequently, participants were instructed to perform the movement and relax after movement completion.

After executing the movement, a 12-second countdown was displayed, during which participants were instructed to relax muscles and place their fingertip back on the sensor box. After the "STOP" cue, the next trial started with a 500ms inter-trial interval (Fig 1). The experiment consisted of 2 experimental blocks of 120 trials, with a seated 90-second rest between blocks.

### Self-reports

Participants were asked to fill out a general questionnaire assessing sociodemographic variables (name, date of birth, sex, civil status, marital status, education, and profession), general health (pregnancy, medical and psychological health problems, medical procedures, treatments and therapy, and pain complaints). This was done to obtain a comprehensive and descriptive overview of our samples. Moreover, patients were asked to fill out additional questions to register LBP related information (i.e., the type [i.e., chronic or recurrent], intensity, duration, localization, of the LBP, current and past treatments). Additionally, all participants were asked to complete Dutch versions of several validated questionnaires.

#### Hospital Anxiety and Depression Scale (HADS)

This is a reliable and valid questionnaire in general and chronic pain populations [38, 39] and screens for anxiety and depression [40]. The HADS is a 14-item scale in which the participants have to report on a 4-point Likert scale the degree to which they have experienced anxious and or depressive feelings over the last week. The scale is divided into two subscales: 7 items for anxiety and also 7 for depression. Higher scores indicate greater levels of depression and anxiety, with scores between 8 and 10 considered as mild, 11 and 14 as moderate and between 15 and 21 as severe, for each subscale.

#### Pain Catastrophizing Scale (PCS)

It is a valid and reliable [41] 13-item scale in which the participants are asked to reflect on previous painful experiences and to indicate their thoughts and feelings when experiencing pain [42]. Responses are given on a 5-point Likert scale ranging from 0 (not at all) to 4 (all the time). Larger scores represent larger catastrophizing behavior levels. Scores are clinically relevant when ≥30. The PCS consists of three subscales: magnification, rumination, and helplessness.

#### Pain Vigilance and Awareness Questionnaire (PVAQ)

This is a valid and reliable scale [43, 44] that consists of 16 items in which participants are asked to report on their vigilance for pain sensations on a Likert scale from 1 (“never”) to 5 (“always”) [45]. The PVAQ consists of two subscales, namely attention to pain and attention to changes in pain. High scores reflect increased levels of hypervigilance to pain sensations.

#### The Roland Morris Disability Questionnaire (RMDQ)

It assesses how daily physical activities and functioning are affected by LBP [46] and it is valid and reliable [47]. Participants answer 24 ‘yes-no’ questions on whether they experienced a specific situation regarding their low back pain that day. The total score ranges from 0 (no disability) to 24 (severe disability) with higher scores indicating higher degrees of LBP related disability.

#### The Tampa Scale of Kinesiophobia (TSK)

This is a valid and sufficiently reliable [48, 49] 17-item questionnaire that measures fear of movement and (re)injury [50]. Items are answered on a 4-point Likert scale ranging from 1 (“strongly disagree”) to 4 (“strongly agree”). A high score on this scale indicates a high degree of kinesiophobia, with a cutoff score of 37.

#### Other self-reports

Participants were asked to indicate to what degree they experienced pain at before and after testing on a VAS scale from 0 to 10.

### Data processing and analyses

#### Self-reports

The total scores at the PVAQ, PCS, and TSK were compared between groups by conducting repeated-measures ANOVA’s and/or t-tests where applicable. Additionally, the total scores on the PCS, PVAQ, and TSK were correlated with the participants’ SEP amplitudes and movement latencies.

#### EEG

Channels were referenced to the average of all electrodes. Next, EEG-signals were filtered with a low cutoff of 1 Hz and a high cutoff of 30 Hz. Afterwards, an automatic artefact rejection was applied to the segments that ranged from -200 to 500 ms around the onset of the tactile stimulus. As a result, all eye movements (including eye blinks and vertical and horizontal eye movements artifacts) were removed via an automatic artifact rejection. Finally, baseline corrections were applied, and the average was calculated for each group. A collapsed localizer was created by averaging the waveforms of all participants [51].

**Fig 2:**
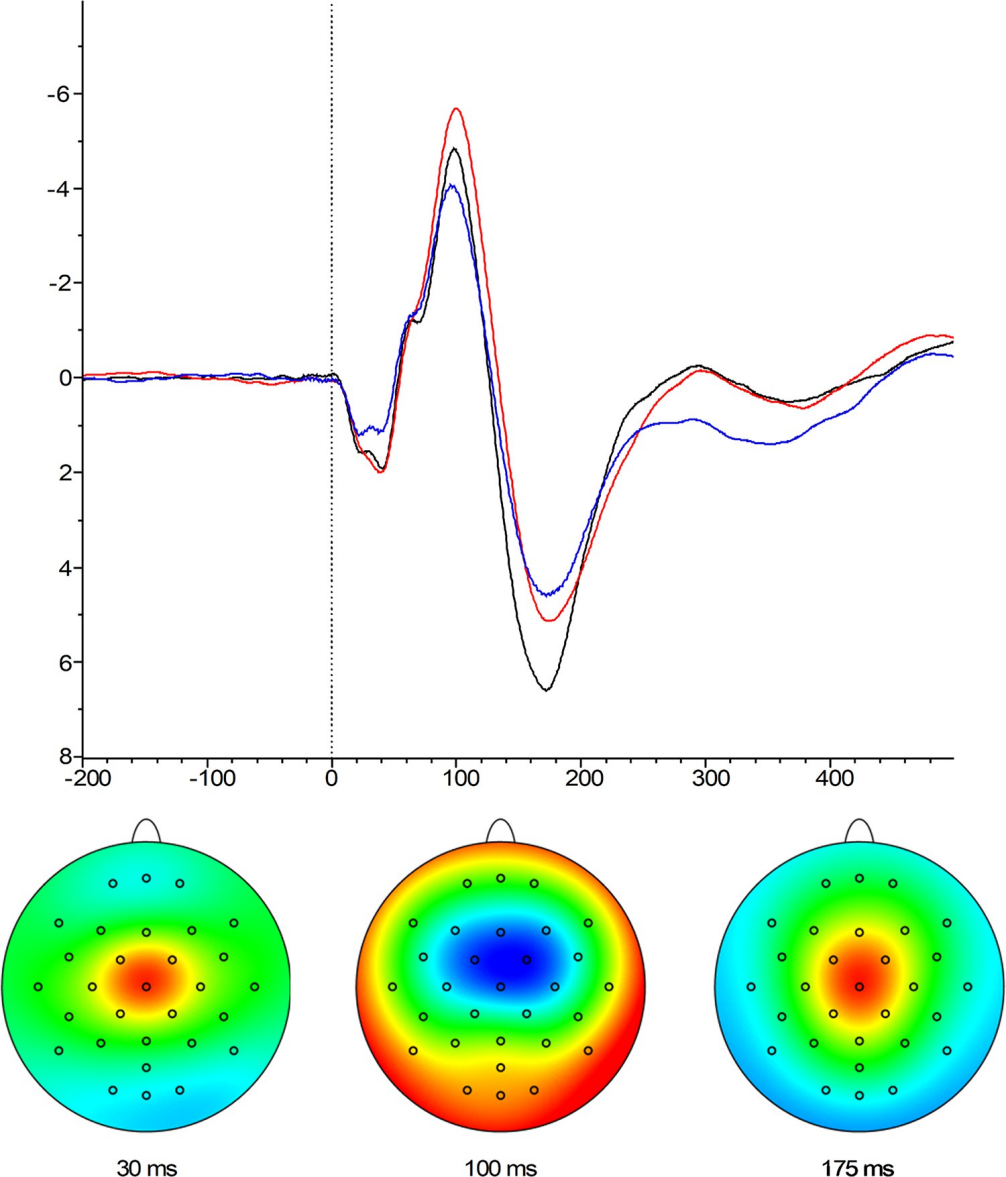
Above: waveforms as presented at the Cz electrode for the three different groups *(red line*: *HC*, *black line*: *CLBP*, *blue line*: *RLBP)*. **Below: topographies at each peak** *(N30*, *N100*, *P175)*.

Based on previous studies [23, 52] and visual inspection, a clear negative peak was detected around 100 ms, and two positive peaks around 30 and 175 ms (see Fig 2). The presence of these peaks was confirmed by calculating the global field power across all participants and conditions. Mean area amplitudes were therefore exported from electrodes FC1, FC2 and CZ for the N30 and P175, and FC1, FC2, Cz and Fz for the N100 component. This area information was extracted from an interval between 20 and 50 ms (N30), 75 and 125 ms (N100), and an interval between 135 and 215 ms (P175). Mean area amplitudes were used because these are known to provide an unbiased measure of amplitude [53]. Comparisons between the participant groups (3 levels) were made by means of a univariate ANOVA, and additional t-tests where applicable. Statistical analyses were performed on the pooled (averaged) channels where the peaks were identified.

#### Reaction latencies

All outliers were removed from the dataset. This was done by eliminating all RTs lower than 100 ms and larger than 2000 ms. Next, all RTs that were faster or slower than 3 times the standard deviation were also removed. Comparisons between the participant groups (3 levels) were made by means of a univariate ANOVA.

## Results

### Participants

The RLBP individuals reported to experience on average 11.30 (*SD* = 9.59) pain flares per year which lasted on average 4.27 (*SD* = 3.33) days per week. They rated on a 0–10 VAS scale a 5.32 (*SD* = 1.89) pain intensity out of 10, and their maximum pain intensity a 6.87 (*SD* = 1.71) out of 10. The duration of the pain free periods ranged across participants between 1 day and several weeks (up to 4 months). Twenty participants have sought non-pharmacological treatment and 19 participants have used pharmacological treatment. Three participants have used other types of treatment. 18 RLBP individuals were single, 7 were co-habiting and 6 were married. Furthermore, 18 were working full time, 3 part-time and 10 were students.

The CLBP individuals on average reported to experience pain on at least 5.72 days (*SD* = 1.38; range = 3–7) per week. On a 0-1- VAS scale, they rated the pain intensity on average 4.27 (*SD* = 1.42) and the maximum intensity 7.11 (*SD* = 1.605). Sixteen participants have sought non-pharmacological treatment and 11 participants have used pharmacological treatment. Two participants reported to have used alternative treatments. 14 CLBP individuals were single, 8 were co-habiting, 3 were married and one other. Moreover, 14 were working full-time, while 3 part-time, 7 were students and 2 were unemployed.

22 HC individuals were single, 9 were co-habiting and 4 were married. Furthermore, 21 HC were working full-time, 6 part-time, 7 were students and one was unemployed. More descriptive data is reported in Table 1.

**Table 1:** Mean demographics and questionnaires data across groups.

	HC	RLBP	CLBP	*p*
***N***	35	32	26	
**gender (*N* female)**	18	17	13	
**age (*N* years)**	36	34	36	*>* .*05*
**righthandedness (*N* left dominant)**	2	2	3	
**education years (*N* years)**	17	16.5	17	*>* .*05*
**back pain at start of testing (*M*(*SD*))**	0.15 (0.48)	1.02 (1.38)	2.32 (1.51)	***<* .*001***
**pain ratings after experiment (M(SD))**	0.58 (1.09)	1.81 (1.73)	4.05 (1.95)	***<* .*001***
**HADS total score (*M*(*SD*))**	5.88(4.82)	8.19 (4.86)	9.92 (5.73)	**.*01***
**HADS depression subscale (*M*(*SD*))**	1.97 (2.42)	2.93 (2.43)	3.00 (2.65)	*>* .*05*
**HADS anxiety subscale (*M*(*SD*))**	3.94 (2.67)	5.25 (3.02)	6.92 (3.64)	**.*002***
**PVAQ total score (*M*(*SD*))**	28.31 (12.88)	33.25 (12.63)	33.31 (13.45)	*>* .*05*
**PCS total score (*M*(*SD*))**	9.89 (8.95)	13.42 (7.22)	14.19 (9.12)	*>* .*05*
**TSK total score (*M*(*SD*))**	31.40 (8.00)	33.84(7.34)	33.08 (7.61)	*>* .*05*
**RMDQ total score (*M*(*SD*))**	.37 (1.40)	4.03 (3.56)	5.69 (2.87)	***<* .*001***

In this table, means and SDs of the descriptive statistics across groups are reported, p values are shown in the last column. HADS, Hospital Anxiety and Depression Scale; PVAQ, Pain Vigilance and Awareness Questionnaire; PCS, Pain Catastrophizing Scale; TSK, Tampa Scale of Kinesiophobia; RMDQ, The Roland Morris Disability Questionnaire. HC, Healthy Controls; RLBP, Recurrent Low Back Pain; CLBP, Chronic Low Back Pain.

### Self-reports

Data from 3 participants were not recorded properly (one from each group) and were therefore excluded from analyses. Table 1 shows the average scores on the questionnaires. For the PCS, PVAQ, and TSK, there were no significant differences between the three groups (*p*’s >.10). The three groups, however, did differ in low back pain before (*F*(2,89) = 25.55, *p* < .001, *ηp*^*2*^ = .36) and after (*F*(2,87) = 35.35, *p* < .001, *ηp*^*2*^ = .45) the experiment. At the start of the experiment, the RLBP reported to experience more LBP compared to the HC (*t*(64) = -3.01, *p* = .009, *d* = -.74). Moreover, the CLBP group reported to have more pain compared to the RLBP (*t*(54.68) = -4.16, *p* < .001, *d* = -1.12) and the HC (*t*(61) = -7.15, *p* < .001, *d* = -1.85). After the experiment, a similar pattern appeared (all *p*’s≤.002).

### EEG

#### N30

The ANOVA revealed no significant effect of group on the N30 amplitudes (*F*(2, 90) = 1.26, *p* = .29, *ηp*^*2*^ = .03).

#### N100

The analysis revealed no significant effect of group on the N100 amplitudes (*F*(2, 90) = 0.86, *p* = .41, *ηp*^*2*^ = .02).

#### P175

The analysis revealed a significant effect of group on the P175 amplitudes (*F*(2, 90) = 78.95, *p* < .001, *ηp*^*2*^ = .64). Further t-tests showed that the CLBP group showed larger amplitudes compared to the HC (*t*(53.27) = 11.39, *p* < .001, *d* = 2.95) and the RLBP group (*t*(56.17) = 10.79, *p* < .001, *d* = 2.85). The HC did not differ significantly from the RLBP group (*t*(63.23) = .41, *p* = .91, *d* = 0.1). SEPs descriptive statistics can be found in the S1 Table.

### Reaction latencies

The data from 3 participants from the CLBP group did not record properly and were therefore excluded from the analyses. A univariate ANOVA revealed no significant differences between the 3 groups (*F*(2,87) = .86, *p* = .43, *ηp*^*2*^ = .02).

### Correlations

No correlations reached significance after application of a Bonferroni correction.

## Discussion

The current study aimed at testing whether CLBP and RLBP patients exhibit stronger somatosensory attentional responses when anticipating back-recruiting movements. During movement preparation, a tactile stimulus was administered to the lower back. EEG SEPs were obtained in response to these stimuli to measure of somatosensory attending. Larger amplitude of the SEPs components indicated increased somatosensory attentional responses. To understand how somatosensory attending may play a role in pain chronicity, SEP amplitudes were compared between CLBP, RLBP, and HC. We hypothesized that individuals suffering from persistent back pain would be more attentive towards their lower back compared to healthy controls during movement preparation, as reflected by increased SEP amplitudes. Since RLBP can be seen as a less severe variant of chronic pain, larger effects were anticipated for the CLBP relative to the RLBP group. Finally, potential associations between SEPs and pain catastrophizing and fear of movement/(re)injury were explored.

Similarly to previous studies [23, 52], we were able to identify three SEP components (N30, N100, P175). Interestingly, the current study only found that the CLBP group had larger P175 amplitudes compared to RLBP and HC, whereas no significant differences were found across groups regarding the N30 and N100 components. Importantly, both the N100 and P175 have been shown to be susceptible to attention, with larger amplitudes when more attention was focused on the tactile event [18, 54–57]. Compatible with the idea of somatosensory attention susceptibility to threat, the N100 SEP has been shown to indicate increased attention towards the specific body part under threat of pain [18]. It is important to note, though, that the latencies of the components in both the current study and our earlier study (N100 vs N120 [6, 7]) may differ due to the different locations of the tactile stimulus on the body. This negative component is thought to represent activity in SII [58] and may therefore reflect attention to a somatosensory stimulus. Our findings, however, do not support this hypothesis, and seem to indicate that individuals with LBP may not be more vigilant towards bodily sensations in the back, a finding that is in line with a previous study [15]. Caution in interpreting this finding is in order, however, considering that the clinical groups in the current study have rather mild symptoms and complaints based on their self-report data. Indeed, the scores on the PCS, PVAQ, and TSK did not differ significantly between the three groups. Perhaps differences can only be observed with more severely disabled chronic pain populations that are recruited from specialized clinical settings rather than the general population.

In contrast with the N100 SEP, the P175 reflects general attention [18, 59]. Possibly, the P175 component may indicate an unspecific effect of threat or arousal in response to the movements, which can arguably be more threatening for the CLBP group compared to the RLBP and HC. Moreover, this component has been suggested to reflect a more complex cognitive or emotional processing of the stimulus [60]. It might therefore be that CLBP individuals were more afraid to perform the movement, and perhaps this might affect how these individuals anticipate and prepare for the movement. Hence, future studies could compare SEPs results with self-reports on fear for the movement.

The three different groups also did not differ on the amplitudes of the early N30 component. This is an interesting component since early sensory components are gated when preparing a limb movement [61], an observation that resembles the phenomenon of sensory suppression [62], i.e. that movements may suppress the processing of sensory information. One might expect, for instance, that if chronic pain populations attend more towards their body part under threat of pain, that these individuals would show smaller amplitudes in these components that indicate sensory suppression. However, the current study is in line with other studies in which the threat of pain did not affect the magnitude of sensory suppression [63].

This study has some limitations. In fact, none of the SEP amplitudes correlated with the scores on the questionnaires. This is surprising since it is known that the expectation of pain motivates people to be vigilant for threats in their body. Especially, if the P175 component may reflect the participants’ aroused state due to the threatening nature of the movements, one might expect that the amplitudes of this specific component would correlate with scores on the TSK. Potentially, the self-report and event-related potentials (ERP) data in the current paradigm may not be sensitive enough to detect individual differences. Furthermore, the fact that there were no between-group differences in these questionnaire scores supports the idea that probably the groups where not distinct enough to capture such effects. Also, the average scores at the RMDQ revealed overall low levels of disability, therefore it might be that patients with more severe conditions were not represented. Future research could use a pre-screening tool to recruit patients and assign them into two subgroups based on their levels of fear of movement and disability. This approach might better capture and distinguish potential differences within the study population. Another limitation of the current study is the considerable number of participants excluded because of recording issues. However, the set-up of this experiment was relatively complex (EEG, sensors and stimulators) and this, together with the strenuous motor task, might have affected the quality of the recorded EEG data. Furthermore, it is known that SEPs might be susceptible to random variation, especially when recorded from the lower back [64], therefore, caution is desirable when drawing conclusion regarding somatosensory processing mechanisms in chronic pain. Therefore, future studies using SEPs in clinical populations might consider assessing the replicability of this outcome in order to obtain a reliable measure of somatosensory processing in chronic pain.

In sum, the current study was unable to show that clinical LBP populations attend more towards their lower back when anticipating a back-posture disturbing movement. Nevertheless, it was found that CLBP individuals showed larger amplitudes to the P175 components compared to both RLBP individuals and HC, which may indicate a higher state of arousal or higher order tactile processing when preparing these movements.

To conclude, this study provides further insights on the mechanisms facilitate the interaction between movement and somatosensory processing. Gaining more knowledge in this field might offer new directions to clinical interventions such as exposure treatment, in which patients are asked to confront pain-evoked movements that they might perceive as threatening. One might speculate that the heightened cortical responses observed in relation to somatosensory processing of this information, might affect the way patients experience these stimuli. It could be interesting, for further studies, to investigate these cortical responses in the context of exposure sessions, in order to examine whether they are predictive of the effectiveness of the exposure treatment or whether these responses evolve during the course of the treatment.

## Supporting information

S1 Checklist Human participants research checklist. (DOCX)

S1 Table SEPs descriptive statistics. CI, confidence intervals; Std. Dev., Standard Deviation; IQR, Interquartile Range. HC, Healthy Controls; RLBP, Recurrent Low Back Pain; CLBP, Chronic Low Back Pain.
(DOCX)
