# Supplementary material for: Does movement preparation enhance attending to bodily sensations in the back in people with persistent low back pain?
Source: PLoS One. 2024 Apr 18;19(4):e0300421. doi: 10.1371/journal.pone.0300421 (PMC11025943; doi:10.1371/journal.pone.0300421)
Supplement: S1 Table — CI, confidence intervals; Std. Dev., Standard Deviation; IQR, Interquartile Range. HC, Healthy Controls; RLBP, Recurrent Low Back Pain; CLBP, Chronic Low Back Pain. (DOCX) [file pone.0300421.s002.docx]

|  | **n30** | | | **n100** | | | **p175** | | |
| --- | --- | --- | --- | --- | --- | --- | --- | --- | --- |
|  | **HC** | **RLBP** | **CLBP** | **HC** | **RLBP** | **CLBP** | **HC** | **RLBP** | **CLBP** |
| **N** | 35 | 32 | 26 | 35 | 32 | 26 | 35 | 32 | 26 |
| **Mean**  **(95% CI)** | 0.88 (0.55 – 1.21) | 0.52 (0.14 – 0.90) | 0.59 (0.22 – 0.97) | -4.09 (-4.82 - -3.36) | -3.29 (-4.29 – -2.29 | -3.74 (-4.73 - -2.76) | 2.90 (2.20 – 3.60) | 2.68 (1.87 – 3.48) | -3.74 (-4.76- -2.72) |
| **Std. Dev.** | 0.96 | 1.06 | 0.93 | 2.12 | 2.77 | 2.43 | 2.04 | 2.24 | 2.53 |
| **IQR** | 1.06 | 0.84 | 1.23 | 2.58 | 3.13 | 2.99 | 2.57 | 2.45 | 3.47 |
| **Range** | 4.41 | 6.16 | 3.93 | 9.91 | 9.59 | 10.63 | 10.07 | 8.667 | 11.032 |
| **Minimum** | -0.51 | -0.894 | -0.99 | -11.15 | -9.22 | -10.06 | -1.08 | -0.84 | -10.49 |
| **Maximum** | 3.89 | 5.25 | 2.93 | -1.25 | 0.36 | 0.57 | 8.99 | 7.83 | 0.55 |

**S1 table. SEPs descriptive statistics**

CI, confidence intervals; Std. Dev., Standard Deviation; IQR, Interquartile Range.

HC, Healthy Controls; RLBP, Recurrent Low Back Pain; CLBP, Chronic Low Back Pain.
